# Risky Sexual Behavior Profiles in Youth: Associations With Borderline Personality Features

**DOI:** 10.3389/fpsyg.2021.777046

**Published:** 2022-01-12

**Authors:** Michaël Bégin, Karin Ensink, Katherine Bellavance, John F. Clarkin, Lina Normandin

**Affiliations:** ^1^École de Psychologie, Université Laval, Quebec, QC, Canada; ^2^Weill Cornell Medical College, Cornell University, New York, NY, United States

**Keywords:** borderline personality disorder, risky sexual behaviors, adolescents, young adults, youth, profiles

## Abstract

Adolescence and young adulthood are peak periods for risky sexual behaviors (RSB) and borderline personality disorder (BPD) features. RSB is a major public health concern and adolescents with BPD may be particularly vulnerable to RSB, but this is understudied. The aim of this study was to identify distinct RSB profiles in youth and determine whether a specific profile was associated with BPD features. Participants were 220 adolescents and young adults (age 14–21) recruited from the community. To identify groups of adolescents and young adults who engage in similar RSB, a latent profile analysis (LPA) was conducted on sexually active youth (57%). Next ANOVA was used to identify how profiles differed in terms RSB dimensions and BPD features. We identified three distinct RSB profiles: (1) a Low RSB profile that was manifested by the majority (77.7%) of youth; (2) an Unprotected Sex in Relationships profile (13.3%) and; (3) an Impulsive Sex Outside Relationships profile (12%) which was manifested by youth with significantly higher BPD features. The findings shed light on the difficulties youth with BPD manifest around integrating sexuality, intimacy, fidelity, and love. This contrasts with the majority of youth who are sexually active in the context of relationships and engage in little or no RSB. The findings have important clinical implications. Adolescent sexuality is frequently in the blind spot of clinicians. To address the elevated risk of RSB in adolescents with BPD, interventions are needed to help adolescents navigate this period and improve their understanding of the reasons for RSB while addressing difficulties in establishing sexual and attachment relationships.

## Introduction

Adolescent risky sexual behavior (RSB) is a major public health concern (Yoon et al., 2018). Adolescence and the transition to adulthood is a critical phase for sexual development and integrating new sexual aspects of self into identity and gaining knowledge and experience that will prepare them for taking on adult social roles, as well as engaging in romantic and sexual relationships (Victor and Hariri, 2016; Suleiman et al., 2017). At puberty onset, clock genes activate the secretion of gonadal steroid hormones, triggering cascades of physical and sexual changes during the maturation of the reproductive system as well as the hypothalamic-pituitary-gonadal axis (HPG). This is associated with the neurobiological remodeling of cortical and limbic circuits (Sisk and Zehr, 2005). Due to this remodeling, puberty and adolescence are particularly vulnerable periods for extreme stressors as well as the emergence of mental illness (Ge et al., 2001; Andersen, 2003; Grant et al., 2003; Turner and Lloyd, 2004; Kessler et al., 2005; Paus et al., 2008). It is also a period characterized by peak risk taking which can pivot development in positive or negative directions (Dahl et al., 2018). Even when adolescents and young adults are aware of the risks, they may find it difficult to translate this knowledge into good sexual decision making “in the heat of the moment” (Victor and Hariri, 2016). From a neurobiological perspective, increased RSB in adolescents and young adults is understood in terms of a triadic model involving adolescence specific decreased threat-related amygdala reactivity to potential negative consequences of risks, increased reward-related ventral striatum reactivity coupled with immature top-down regulation from the prefrontal cortex (Victor and Hariri, 2016). However, findings from other studies suggest that only a subgroup of youth with high reward-related ventral striatum reactivity to sexually stimulating material showed elevated levels of RSB and may be particularly at risk (Demos et al., 2012).

### Sexuality and Risky Sexual Behaviors During Adolescence and Young Adulthood

Becoming sexually active over the adolescent and young adulthood period is normative, with 30% of 15–17 year-olds, 68% of the 18–20 year-olds, and 86% of 20–24 year-old Canadians reporting being sexually active (Roterman, 2012). The majority of youth have their first sexual experience with someone they are going steady with, suggesting that it is normative to integrate sexuality and romantic relationships (Abma and Martinez, 2017). A certain degree of risk appears to be widespread with 46% of youth reporting not using protection during first intercourse (Abma and Martinez, 2017). Risky sexual behaviors (RSB) peak in late adolescence and early adulthood (Victor and Hariri, 2016; Dahl et al., 2018) and this age group has the highest rates of sexually transmitted infections (STIs) (Weinstock et al., 2004; Shannon and Klausner, 2018), unplanned pregnancies (Finer, 2010), and intimate partner violence (Stöckl et al., 2014). Other negative consequences include damage to relationships and reputation, family conflicts, and financial and legal problems (Turchik and Garske, 2009; Crandall et al., 2018).

Adolescent risk taking occurs in the context of a matrix of risk and protective factors at the social and family level, as well as at an individual level. Protective factors include open family communication and appropriate monitoring, investment in school and activities, and access to information, contraception, and condoms (Arbel et al., 2018). Risk factors include disadvantage and violence such as family conflict and sexual abuse (Abajobir et al., 2017), as well as adolescent alcohol and substance abuse (Centers for Disease Control and Prevention [CDC], 2018), insecure attachment (Kim and Miller, 2020), early puberty (Deardorff et al., 2005), and earlier age of first sexual relationships (Edgardh, 2000, 2007). In addition, proximal risk factors include parental unresponsiveness to adolescent worries and support seeking which have been shown to increase same or next day RSB (Arbel et al., 2018). At an individual level, adolescents with mental health problems are at heightened risk of RSB and there is some preliminary evidence that BPD features are associated with increased risk of RSB. For this reason, we were particularly interested in examining whether adolescents with high RSB of particular kinds showed high BPD features.

### Borderline Personality and Adolescence

BPD is characterized by instability in interpersonal relationships, emotional dysregulation, marked impulsivity, and unstable self-image (APA, 2013). Recent research supports the validity of the BPD diagnosis during adolescence (Chanen and Kaess, 2012; Stepp, 2012; Fossati, 2014; Chanen, 2015; Sharp and Fonagy, 2015; Sharp, 2016a,b). In adolescents from the community, the prevalence of BPD is estimated at 3% (Lewinsohn et al., 1997; Johnson et al., 2008; Leung and Leung, 2009; Zanarini et al., 2011). BPD traits peak during adolescence and tend to decline during the mid- to late-20s (Cohen et al., 2005; Miller et al., 2008; Bomovalna et al., 2009; Stepp et al., 2014; Winsper et al., 2016). A dimensional approach to adolescent BPD is increasingly adopted as BPD appears to be a dimension rather than a taxon (Haslam et al., 2012) especially amongst youth.

### Borderline Personality Disorder and Risky Sexual Behaviors in Youth

There are few studies of RSB and BPD in adolescents and young adults (Penner et al., 2019). In adults, BPD features are related to RSB (Sansone and Sansone, 2011). For example, adults with BPD are more susceptible to engage in impulsive sexual behaviors with people they do not know well (Sansone and Wiederman, 2009) and report more sexual partners overall (Bouchard et al., 2009; Sansone et al., 2011a,b).

Similarly, adolescents with BPD have a higher number of sexual partners (Lavan and Johnson, 2002; Thompson et al., 2017), more unsafe partners (Thompson et al., 2017), and higher rates of STIs (Lavan and Johnson, 2002; Chanen et al., 2007). In the longitudinal Pittsburgh Girls study, adolescent girls with early symptoms of BPD were at heightened risk for the development of adolescent RSB, while the reverse association did not hold (Choukas-Bradley et al., 2020). In another longitudinal study, sending and receiving sexual text messages, or “sexting” at age 16 predicted BPD features at age 18, suggesting that certain types of sexual behaviors may be more specifically associated with early BPD trajectories (Brinkley et al., 2017). There are also divergent findings. For example, no BPD-related differences in RSB were found in a psychiatric inpatient sample, although adolescents with BPD were less self-affirmative regarding refusing sexual pressure (Penner et al., 2019). Another study reported mixed results with higher rates of STIs amongst adolescents with BPD, but no significant differences in RSB frequency (Chanen et al., 2007). These studies have limitations in that they focused only on females. Furthermore, some of these studies did not use optimal methodologies for identifying RSB profiles associated with adolescent BPD.

### Mechanisms Linking Borderline Personality Disorder and Risky Sexual Behaviors

Few studies have examined the mechanisms underlying the association between BPD and RSB and none did amongst adolescents. In adults, substance abuse in individuals with BPD is associated with more sexual partners, representing one possible mechanism (Chen et al., 2007; Harned et al., 2011). BPD-associated impulsivity is thought to increase the risk of impulsive RSB (Rickards and Laaser, 1999; Kalichman and Rompa, 2001; Jardin et al., 2017). Furthermore, sexual compulsivity as a way of trying to deal with BPD-associated negative affectivity has been argued to underlie RSB (Reid et al., 2011). There may be other underlying mechanisms that are particularly relevant for understanding BPD-related RSB in youth during this key transitional period of initiation of sexual and romantic relationships, and of development of sexual identity. For example, insecure and especially anxious attachment (Kim and Miller, 2020), low self-esteem, difficulties in establishing a dating identity (Kerpelman et al., 2013) have been shown to be associated with more RSB.

### Dimensional and Categorical Approaches to Risky Sexual Behaviors

An important issue in understanding how RSB relate to BPD features concerns how RSB is measured i.e., whether it is measured using a dimensional approach or one that is based on different profiles or types of RSB. RSB is often conceptualized and measured as a continuum of severity with the assumption that frequency of behaviors reflect severity. This approach has important disadvantages. RSB covers a wide array of behaviors, but when aggregate scores are used, all types of RSB are treated as equivalent. Furthermore a dimensional approach which aggregates the number of RSB behaviors makes it impossible to distinguish different profiles of RSB and thus to investigate their differential associations with risk factors, psychopathology and development (Turchik and Garske, 2009). For example, some sexual risk taking behaviors such as unprotected sex occur in the contexts of committed relationships, others are more impulsive and involve a higher number of partners and are less compatible with intimate relationships (Turchik and Garske, 2009).

Few studies have addressed the question of how to conceptualize RSB. While one study failed to find RSB subgroups and concluded that RSB is a dimension rather than a taxon (Marcus et al., 2011), others found that RSB can be divided into five factors that better reflect different patterns of RSB (Turchik and Garske, 2009). An important limitation of both these studies is that they used variable centered analysis while person centered analyses are much more suited to identifying groups of individuals with similar profiles (see Berzenski and Yates, 2011 for a comprehensive overview of person-centered approaches). To date no studies have used a person-centered methodological approach such as latent profiles analyses (LPA). This approach makes it possible to examine whether specific RSB profiles are related to BPD.

### The Current Study

To address the gaps in the literature regarding whether distinct RSB profiles can be identified, and are associated with BPD, the objectives of the present study were to: (1) examine RSB profiles in adolescents and young adults (aged 14–21) from a community sample using LPA; and (2) investigate whether specific profiles were associated with significantly higher BPD features.

We hypothesized that it would be possible to identify (a) a large group presenting a profile of engaging predominantly in normative sexual activity and little RSB; and (b) one or more profiles with some RSB including one group presenting RSB outside committed romantic relationship. It was hypothesized that BPD features would be related to specific types of RSB such as engaging in impulsive sexual behaviors and having more uncommitted partners.

## Methodology

### Participants

Participants were 220 adolescents and young adults (age 14–21) from a community sample. 114 were aged 14–17 whereas 106 were aged 18–21. Of the 220, 77.7% were female and 22.3% were male. To be included in the final sample, they had to be sexually active during the past 6 months. This resulted in a final sample of 126 (82% female and 17.5% male) adolescents and young adults (age 14–21, *M* = 18.8, *SD* = 2.32) for the final analyses, constituting 57% of the initial sample. Females and males were as likely to have been sexually active within 6 months prior to the study. Of the 126 participants, 41 were aged 14–17 (36% of the adolescents) and 85 were 18–21 (80.2% of the young adults).

### Procedure

Participants were recruited at high schools in Quebec City and surroundings areas, as well as at a university in the same Canadian city. The study, the objectives, and the procedure to participate were presented in class or via an email list. After they were presented with the online consent form, they were invited to complete a series of questionnaires online on a variety of topics such as sexuality, personality and related difficulties, psychiatric symptoms, and interpersonal functioning. This study is part of a broader research project on personality disorders amongst adolescents and young adults.

### Ethical Approval and Informed Consent

The consent provided by the adolescents was in accordance with Article 21 of the Civil Code of Québec which specifies that from age 14, adolescents can decide to consent to certain activities such as participating in research. Furthermore, respondents completed a consent form that clearly stated that the researchers have an obligation to report sexual and physical abuse situation. This study was approved by the Ethics Committee of Laval University.

### Instruments

#### Sexual Risk Survey

The SRS is a 23-item questionnaire divided into five scales, namely *Sexual Risk Taking With Uncommitted Partners, Risky Sex Acts* (non-protected sex or under the influence of a substance), *Impulsive Sexual Behaviors*, *Risky Anal Sex*, and *Intent to Engage in Sexual Behaviors* (Turchik and Garske, 2009). For each item, the participants reported the number of times they engaged in the behavior in the past 6 months. Then, the frequencies are recoded on an ordinal scale from 0 to 4. A higher score reflects a higher frequency for a given item. The Internal consistency for each scale is good and is ranging from 0.78 to 0.89 (Cronbach’s α) except for *Risky Anal Sex* for which it was poorer (α = 0.61). For the current study, in French translation of the measure was used that previously showed good psychometric properties (Laberge, 2013).

#### Borderline Personality Features Scale for Children

The BPFS-C (Crick et al., 2005) is a 24-item questionnaire rated on a five-point Likert scale (*Never true* to *Always true*) assessing four main features of borderline personality dimensionally, namely *Affective instability*, *Identity problems*, *Negative Relationships*, and *Self-Harm*. A total score of borderline features ranging from 24 to 120 is calculated from the 24 items. A higher score is indicative of more borderline features. The BPFS-C presents with an adequate internal consistency with Cronbach’s alphas ranging from 0.76 to 0.89 across scales in a community sample (Sharp et al., 2011). The criterion validity of the BPFS-C has been previously evaluated and a cutoff score of 66 for the presence of borderline personality disorder (BPD) has been suggested (Chang et al., 2011). Furthermore, the French version of the BPFS-C has also been shown to have good internal consistency with a Cronbach alpha of 0.91 for the total score (Ensink et al., 2019).

### Statistical Analyses

To identify groups of adolescents and young adults with similar patterns of RSB, a latent profile analysis (LPA) was conducted. The fit of the mixture model (LPA) was tested using Mplus 8.6 (Muthén and Muthén, 1998–2017). Models including two to five profiles were evaluated. Four fit indices were used to select the best fitting model. The selection was based on the lowest Akaike information criterion (AIC) (Akaike, 1974) and Bayesian information criterion (BIC) (Schwarz, 1978) which assess model fit with varying degrees of consideration for parsimony, the highest entropy which represents the percentage of participants correctly classified by the model (Ramaswamy et al., 1993) and the Lo-Mendell-Rubin adjusted likelihood ratio (LMRT) (Lo et al., 2001) which evaluated whether the model fits the data significantly better than a model with *k–1* profiles, that is to say a model with one less profile. Furthermore, it has been suggested that the BIC is the most reliable measure to assess model fit (Nylund et al., 2007). Given the number of profiles, the unequal profile sizes but a strong separation between the profiles, the actual statistical power for the analyses (BLRT) could be estimated between 0.72 and 0.90 based on a Monte Carlo simulation study and power curves with *N* = 100 and *N* = 150 (Dziak et al., 2014). Then, ANOVAs were conducted with Tamhane’s T2 or LSD *post hoc* tests depending on the assumption of homoscedasticity to examine on what scales the profiles differed from each other. One-way ANOVAs and LSD *post hoc* tests were also used to determine whether the groups were different in terms of borderline personality pathology. The ANOVAs were conducted with SPSS 24 using an alpha threshold of 0.05 with a conservative Bonferroni correction for the number of tests. The final alpha threshold used was therefore 0.01 after the applied correction for the ANOVAs and 0.05 for multiple comparisons for which a correction is already applied.

## Results

The best fitting model was a three-profile solution (see Table 1). Compared to a two-profile solution, it showed lower AIC and BIC. The LMRT indicated that a third profile provided a better fit compared to the two-profile solution. Also, the four-profile model showed a lower AIC, but a higher BIC. The LMRT indicated that the fourth profile did not add extra information in explaining the associations between the participants in terms of RSB. Finally, the five-profile solution showed an increase of both the AIC and BIC as an indication of a deteriorating fit up to that point with the addition of more profiles. The entropy for two to five profiles was equal across all models and was considered excellent. The final model included three profiles with 98, 13, and 15 participants, respectively.

**TABLE 1:** Fit statistics for potential LPA models (*N* = 126).

Profiles	AIC	BIC	LMRT (*p*)	Entropy
2 profiles	995.51	1040.89	0.007	0.983
3 profiles	**834.11**	**846.94**	**0.037**	**0.985**
4 profiles	824.06	883.48	0.384	0.986
5 profiles	839.67	893.10	0.255	0.984

*AIC, Akaike information criterion; BIC, Bayesian information criterion, and LMRT, Lo-Mendell-Rubin Likelihood Ratio test. Bold represents optimal solution.*

To examine what characterized each profile in terms of RSB, ANOVAs were conducted using the five scales of the SRS. Because of the unequal number of participants between groups, the assumptions of the normality of the distribution of the errors, the independence of observations, and the homogeneity of the variances were thoroughly examined. For some scales (Risky anal sex, Impulsive sexual behaviors, Sexual risk taking with uncommitted partners), the assumption of homogeneity of the variances could not be met. Therefore, the *p*-value of the Welch’s *F-*test was used instead of the *p-*value of the Fischer’s *F-*test as it is robust to the violation of this assumption (Moder, 2007, 2010). The ANOVAs revealed at least one significant difference between the three identified profiles on the Risky anal sex [*F*_(2, 121)_ = 12.64, *p* = 0.01], Risky sex acts [*F*_(2, 119)_ = 9.00, *p* = 0.01], Impulsive sexual behaviors [*F*_(2, 122)_ = 21.69, *p* < 0.001], and Sexual risk taking with uncommitted partners [*F*_(2, 122)_ = 27.27, *p* < 0.01] scales. No differences were found between the profiles regarding the Intent to engage in sexual behaviors [*F*_(2, 122)_ = 5.04, *p* = 0.137]. The full results are presented in Table 2.

**TABLE 2:** ANOVAs with the three profiles as independent variables and the five SRS scales as dependent variables.

		Sum of squares	df	Mean square	F	*p*
Risky anal sex	Between groups	3.82	2	1.91	12.64	0.01
	Within groups	18.27	121	0.15		
Risky sex acts	Between groups	2.52	2	1.26	9.00	<0.001
	Within groups	16.66	119	0.14		
Impulsive sexual behaviors	Between groups	12.13	2	6.07	21.69	0.001
	Within groups	34.11	122	0.28		
Intent to engage in sexual behaviors	Between groups	3.43	2	1.67	5.04	0.137
	Within groups	40.47	122	0.33		
Sexual risk taking with uncommitted partners	Between groups	19.90	2	9.95	27.27	<0.001
	Within groups	44.52	122	0.37		

**FIGURE 1:**
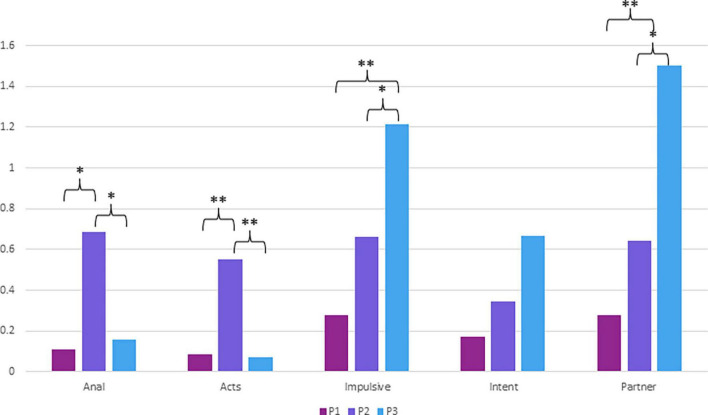
*Post hoc* multiple comparisons tests (Tamhane’s T2 and LSD) using the five scales of the SRS. P1 (*N* = 98); P2 (*N* = 13); P3 (*N* = 15); Anal (Risky anal sex); Acts (Risky sex acts); Impulsive (Impulsive sexual behaviors); Intent (Intent to engage in sexual behaviors); Partner (Sexual risk taking with uncommitted partners); *p < *0.05*; ^**^p < 0.01. Tamhane’s T2 tests were used when the assumption of homoscedasticity was not met.

Multiple comparisons tests revealed that participants in Profile 2 reported significantly more risky anal sex behaviors than those in Profile 1 (*p* = 0.013) and Profile 3 (*p* = 0.035). Profile 1 and 3 did not differ significantly in terms of risky anal sex (*p* = 0.897). Participants in Profile 2 also reported significantly more risky sexual acts than those in Profile 1 (*p* = 0.001) and in Profile 3 (*p* = 0.003). However, Profiles 1 and 3 showed no significant difference (*p* = 0.989). In terms of impulsive sexual behaviors, Profile 3 showed significantly higher scores than both Profile 1 (*p* = 0.001) and Profile 2 (*p* = 0.019) whereas the two latter profiles did not differ significantly (*p* = 0.132). Regarding sexual risk taking with uncommitted partners, Profile 3 had significantly higher scores than Profile 1 (*p* = 0.001) and Profile 2 (*p* = 0.017). Finally, no differences were detected between the profiles in terms of intent to engage in sexual behaviors. In summary, Profile 1 was characterized by low risky sexual behaviors on all scales whereas Profile 2 was characterized by higher scores on risky anal sex as well as risky sex acts, but within committed relationships or known partners, and profile 3 by impulsive sexual behaviors and sexual risk taking with uncommitted partners. The results are presented graphically in Figure 1.

Next, we wanted to examine how these profiles were associated with BPD features. A one-way ANOVA was conducted to examine potential differences between the profiles on borderline personality pathology. The results are presented in Table 3. The results revealed at least one significant difference between the profiles [*F*_(2, 123)_ = 5.03, *p* = 0.008]. LSD multiple comparisons tests showed that participants of Profile 3 had significantly more pathological levels of borderline personality pathology than both Profile 1 (*p* = 0.002) and Profile 2 (*p* = 0.049). No significant difference was detected between Profile 1 and Profile 2 (*p* = 0.674).

**TABLE 3:** ANOVA with the three profiles as independent variables and borderline personality features as the dependent variable.

Profiles	N	Mean	*SD*	Minimum	Maximum	*N* > 66
1	98	48.68	14.42	22.00	93.00	10
2	13	50.46	15.75	35.00	89.00	1
3	15	61.23	11.69	44.00	84.00	5

	**Sum of squares**	**df**	**Mean square**	***F***	***p***	

Between groups	2048.96	2	1024.48	5.03	0.008	
Within groups	25059.36	123	203.65			

## Discussion

The aim of the study was to identify distinct RSB profiles in adolescents and young adults and to examine the association between RSB profiles and BPD features. In terms of descriptive findings pertinent to our study, 57% of the study participants were sexually active; 36% in the 14–17 age group and 85% in the 18–21 age group. The findings of the percentage of sexually active adolescents and young adults are broadly in line with that of previous research with a Canadian sample showing that 30% of the 15–17 age group, 68% of the 18–20 age group and 86% of the 20–24 group were sexually active (Roterman, 2012).

In terms of our main findings and consistent with our hypotheses, we identified three distinct RSB profiles in sexually active youth including a profile with low RSB, another with RSB mainly within the context of committed relationships and another profile with RSB outside romantic relationships associated with higher levels of BPD features. We named these profiles the Low Risk profile, the Unprotected Sex in Relationships profile and an Impulsive Sex Outside Relationships profile and discuss them in detail below.

### Low Risky Sexual Behaviors Profile

In line with our hypotheses most sexually active youth (77.7%) manifested a Low RSB profile characterized by low to no RSB on the five SRS scales. Adolescents with this profile had significantly lower levels of BPD features in comparison to the Impulsive Sex Outside Relationships profile. This suggests, as proposed by Harden (2014) that consensual sexual activities in adolescents and young adults are developmentally normative and potentially healthy and that youth have the developmental capacity to regulate the health risks inherent in sexual activity. The relationship dimension of sexual experience may be a critical moderator of its psychological impact (Harden, 2014) given that adolescence and young adulthood are considered key periods for the development of sexual identity and the integration of sexuality into romantic relationships in preparation for mature adult social roles. Our findings are thus contrary to the risk perspective which portrays adolescence and young adulthood as a period marked by neurobiological immaturity in judgment and vulnerabilities to reward that compromise the capacity to have safe sexual relationships.

Our findings that only 36% of adolescents aged 14–17 reported being sexually active suggest that it is be less normative for younger adolescents to be sexually active.

### Unprotected Sex in Relationship Profile

The Unprotected Sex in Relationships profile was manifested by 10.3% of youth and represents a subgroup of youth who engage in RSB mostly in the form of unprotected anal, vaginal, or oral sex. However, compared to the Impulsive Sex Outside Relationship profile, this profile showed significantly lower levels of impulsive sexual behavior or sexual risk taking with uncommitted partners, suggesting that unprotected sex occurs mostly in committed relationships. Adolescents with this profile also had significantly lower levels of BPD features compared to those in the Impulsive Sex Outside Relationship profile.

Our findings are broadly consistent with that of the Non-Use of Contraception of Canadian Youth in the 2009–2010 Canadian Health Survey (Dunn et al., 2019) which showed that 15% of youth had unprotected sex. It is also in line with previous findings that condom use is lower in the context of sex with regular partners in adults (Macaluso et al., 2000; Fetner et al., 2020). It may be that youth view unprotected sex regular partners as involving lower risk of HIV/STDs and pregnancy. However, we cannot rule out the alternative hypothesis that youth perceive a greater risk (e.g., damage to the relationship, arguments, lack of sex) in discussing and insisting on condom use for STD/pregnancy prevention compared to the risk of contracting an STD because of non-condom use.

Further research is also needed to determine whether youth in this group used other forms of contraception as the use of oral contraception was associated with decreased condom use by youth in the 2009–2010 Canadian Health Survey (Dunn et al., 2019) as well as other studies (Corbin and Fromme, 2002; Parkes et al., 2009). Adolescents may think that in the context of romantic relationships where contraception is used, protection is not necessary. However, this group is at elevated risk of STIs as poor condom use is the most important risk factor for STIs (Baldwin et al., 2004; Hsu et al., 2015) followed by frequency of intercourse (Baldwin et al., 2004), and not the number of sexual partners (Partridge and Koutsky, 2006). Adolescents in this group also engaged in significantly more unprotected anal intercourse which carry an increased risk of HIV and STDs compared to unprotected vaginal intercourse, particularly for women (Jenness et al., 2011). In sum, youth in this group displayed some level of RSB in the form of unprotected sex, but their sexuality appears to be less chaotic and involved fewer partners than participants in the Impulsive Sex Outside Relationship profile. The fact that this group of adolescents did not engage in more impulsive sex, but that their RSB manifested as non-use of protection in relationships would suggest that other factors such a belief, knowledge and their or their partner’s preferences regarding use of protection may enter into their weighting of risk vs. the benefits of having uninterrupted sex. Compared to adults, where 70% did not use protection, our finding that show that only 10.3% of adolescents did not use protection should also be considered in the light of findings that 70% of adult Canadians did not use protection, suggesting that it is not a behavior specific to youth and should be understood in terms of perceived risk, preferences, and knowledge.

### The Impulsive Sex Outside Relationship Profile and Borderline Personality Disorder Features

The Impulsive Sex Outside Relationship profile displayed by 12% of youth was characterized by significantly higher levels of impulsive sexual behaviors as well as higher levels of sex with uncommitted partners than the other two profiles, but a low level of unprotected sex. This group also had significantly higher BPD features compared to the other profiles. Our findings indicate that RSB characterized by impulsive sex outside committed romantic and attachment relationships are associated with BPD features in youth. This extends previous findings that adults with BPD are more susceptible to engaging in impulsive sexual behaviors with people they do not know well (Sansone and Wiederman, 2009) and report more sexual partners overall (Bouchard et al., 2009; Sansone et al., 2011a,b). It is also consistent with previous findings that adolescents with BPD have a higher number of sexual partners (Lavan and Johnson, 2002; Thompson et al., 2017).

Many studies have shown that RSB is related to general impulsivity (Charnigo et al., 2012; Dir et al., 2014) regardless of gender. Our findings add an important nuance to current knowledge in this area, as youth with this profile demonstrated impulsivity specifically regarding sex, but this did not extend to non-use of protection. At this stage it is not clear whether the higher number of partners and impulsive sex are driven by the desire to have sex, sensation seeking and sexual compulsivity, or failed attempts at forming relationships. Identity diffusion and unstable and stormy interpersonal relationships are features of BPD and youth with BPD features may have difficulties developing stable dating relationships. In sum, it may be that the desire for sex or the desire for connection and intimacy with someone, which are normal developmental objectives, drive the findings of more sexual engagement with uncommitted partners. Put differently, youth who are unable to develop relationships with committed partners may resort to sex with multiple partners to satisfy needs for sex, intimacy, and connectedness. However “hooking up” in this way is associated with high mental health costs and may be psychologically destabilizing and is associated with increased depressive symptoms (Mendle et al., 2013). Helping adolescents and young adults with BPD features to understand their behavior in this way and to develop the capacity to maintain stable intimate relationships could potentially reduce this type of risk-taking behavior.

### Strengths and Limitations of the Study

This study has several strengths. It is one of few studies examining the relationship between RSB and BPD amongst adolescents and young adults. It used LPA, a person-centered state of the art methodology to identify youth’s different RSB profiles. The use of LPA is an important step forward from previous methodologies using aggregate scores of RSB, as it makes it possible to identify groups of adolescents who engage in distinct types of RSB and have specific RSB profiles. This in turn makes it possible to identify RSB profiles associated with psychopathology. In sum it makes possible to have a more fine-grained understanding of RSB. Also, while most studies focused on psychiatric patients, the current study focused on participants from the community, thus extending knowledge in the field to an important population and making the results more applicable to understanding adolescent RSB and links with BPD features. In addition, the sample included male participants, addressing a limitation of all previous studies who focused only on females. Furthermore, we used a dimensional measure of BPD features in line with current thinking regarding BPD in youth (Haslam et al., 2012; Stepp, 2012; Rothschild et al., 2013).

The findings must also be interpreted in the light of some limitations. The sample size was relatively small for the LPA, but it is sufficient for examining a limited number of variables, a small number of profiles given little to no overlap between the profiles (Muthén and Muthén, 1998–2017), as is the case in the current study. Another limitation concerns the cross-sectional nature of the data so that it was not possible to examine longitudinal trajectories of RSB in relation to trajectories of BPD features. Longitudinal studies are needed to better understand how both constructs evolve from early adolescence to young adulthood and investigate the hypothesis that specific types of RSB are specific to a group of youth with a higher BPD features peak that does not decline in early adulthood. Furthermore, while males were also included in the study, the sample remained predominantly female. This has the advantage of making our findings more comparable to most samples which are exclusively female. Unfortunately, the number of male participants was small and made it impossible to examine gender differences in RSB. It is evident that studies with a more balanced male-to-female ratio are needed. Furthermore, in this study we did not address other factors known to be associated with an increased risk of RSB such as histories of childhood sexual abuse, family disorganization, insecure attachment, delinquency, and substance abuse.

## Conclusion

The study findings indicate that most adolescents and young adults do not manifest RSB or manifest them in the context of relationships. Adolescents with BPD features are particularly at risk of engaging in impulsive sex with multiple partners reflecting difficulties in integrating sexuality, intimacy, fidelity, and love. The findings have important clinical implications. Sexuality is often ignored in clinical work with adolescents. However, being alert to RSB in clinical work with adolescents and young adults with BPD and helping them overcome their difficulties in establishing sexual and attachment relationships may reduce the risk of resorting to impulsive sex with multiple partners.

## Data Availability Statement

The raw data supporting the conclusions of this article will be made available by the authors, without undue reservation.

## Ethics Statement

The studies involving human participants were reviewed and approved by the Comité d’Éthique à la Recherche de l’Université Laval (CERUL). Written informed consent from the participants’ legal guardian/next of kin was not required to participate in this study in accordance with the national legislation and the institutional requirements.

## Author Contributions

MB was responsible for the co-writing of the “Introduction” and “Discussion” section, conducted the statistical analyses and wrote the “Methodology” and “Results” section. KE and LN were responsible for the elaboration of the broader research project and the data collection, and co-wrote the introduction and the discussion. KB was responsible for the original ideas, the elaboration of the objectives and hypotheses, the literature review, and co-wrote the introduction. JC was responsible for the integration of the research project into the broader theoretical framework which the article is rooted into and co-wrote the introduction and the discussion. All authors contributed to the article and approved the submitted version.

## Conflict of Interest

The authors declare that the research was conducted in the absence of any commercial or financial relationships that could be construed as a potential conflict of interest.

## Publisher’s Note

All claims expressed in this article are solely those of the authors and do not necessarily represent those of their affiliated organizations, or those of the publisher, the editors and the reviewers. Any product that may be evaluated in this article, or claim that may be made by its manufacturer, is not guaranteed or endorsed by the publisher.

## References

[B1] Abajobir A. A. Kisely S. R. Maravilla J. C. Williams G. Najman J. M. (2017). Gender differences in the association between childhood sexual abuse and risky sexual behaviours: a systematic review and meta-analysis. *Child Abuse Neglect* 63 249–260. 10.1016/j.chiabu.2016.11.023 27908449

[B2] Abma J. C. Martinez G. M. (2017). Sexual activity and contraceptive use among teenagers in the United States, 2011–2015. *Natl. Health Statisti. Rep.* 104 1–23. 28696201

[B3] Akaike H. (1974). A new look at the suicidal model identification. *IEEE Transac. Automat. Control* 19 716–723.

[B4] Andersen S. L. (2003). Trajectories of brain development: point of vulnerability or window of opportunity? *Neurosci. Behav. Rev.* 27 3–18. 10.1016/S0149-7634(03)00005-8 12732219

[B5] APA (2013). *Diagnostic and Statistical Manual of Mental Disorders* 5th Edn. Arlington: American Psychiatric Publishing.

[B6] Arbel R. Perrone L. Margolin G. (2018). Adolescents’ daily worries and risky behaviors: the buffering role of support seeking. *J. Child Adolesc. Psychol.* 47 900–911. 10.1080/15374416.2016.1169536 27379707 PMC6193855

[B7] Baldwin S. B. Wallace D. R. Papenfuss M. R. Abrahamsen M. Vaught L. C. Giuliano A. R. (2004). Condom use and other factors affecting penial human pappillomavirus detection in men attending a sexually transmitted disease clinic. *J. Am. Sex. Trans. Dis. Assoc.* 31 601–607. 10.1097/01.olq.0000140012.02703.10 15388997

[B8] Berzenski S. R. Yates T. M. (2011). Classes and consequences of multiple maltreatment: a person-centered analysis. *Child Maltreat.* 16 250–261. 10.1177/1077559511428353 22146858

[B9] Bomovalna M. A. Hicks B. M. Iacono W. G. McGue M. (2009). Stability, change, and heritability of borderline personality disorder traits from adolescence to adulthood: a longitudinal twin study. *Dev. Psychopathol.* 21 1335–1353. 10.1017/S0954579409990186 19825271 PMC2789483

[B10] Bouchard S. Godbout N. Sabourin S. (2009). Sexual attitudes and activities in women with borderline personality disorder involved in romantic relationships. *J. Sex Mar. Ther.* 35 106–121. 10.1080/00926230802712301 19266380

[B11] Brinkley D. Y. Ackerman R. A. Ehrenreich S. E. Underwood M. K. (2017). Sending and receiving text messages with sexual content: relations with early sexual activity and borderline personality features in late adolescence. *Comput. Hum. Behav.* 70 119–130. 10.1016/j.chb.2016.12.082 28824224 PMC5560614

[B12] Centers for Disease Control and Prevention [CDC] (2018). *2016 Sexually Transmitted Diseases Surveillance.* Atlanta, GA: Centers for Disease Control and Prevention.

[B13] Chanen A. M. (2015). Borderline personality disorder in young people: are we there yet? *J. Clin. Psychol.* 71 778–791. 10.1002/jclp.22205 26192914

[B14] Chanen A. M. Jovev M. Jackson H. J. (2007). Adaptive functioning and psychiatric symptoms in adolescents with borderline personality disorder. *J. Clin. Psychiatry* 68 297–306. 10.4088/JCP.v68n0217 17335330

[B15] Chanen A. M. Kaess M. (2012). Developmental pathways to borderline personality disorder. *Curr. Psychiatry Rep.* 14 45–53. 10.1007/s11920-011-0242-y 22009682

[B16] Chang B. Sharp C. Ha C. (2011). The criterion validity of the Borderline Personality Feature Score for Children in an adolescent inpatient setting. *J. Pers. Disord.* 25 492–503. 21838564 10.1521/pedi.2011.25.4.492

[B17] Charnigo R. Noar S. M. Garnett C. Crosby R. Palmgreen P. Zimmerman R. S. (2012). Sensation seeking and impulsivity: combined associations with risky sexual behavior in a large sample of young adults. *J. Sex Res.* 50 480–488. 10.1080/00224499.2011.652264 22456443 PMC4520301

[B18] Chen E. Y. Brown M. Z. Lo T. T. Y. Linehan M. M. (2007). Sexually transmitted disease rates and high-risk sexual behaviors in borderline personality disorder versus borderline personality disorder with substance use disorder. *J. Nervous Ment. Dis.* 195 125–129. 10.1097/01.nmd.0000254745.35582.f6 17299299

[B19] Choukas-Bradley S. Hipwell A. E. Roberts S. R. Maheux A. J. Stepp S. D. (2020). Developmental Trajectories of Adolescent Girls’ Borderline Personality Symptoms and Sexual Risk Behaviors. *J. Abnor. Child Psychol.* 48 1649–1658. 10.1007/s10802-020-00699-4 32918189 PMC7857542

[B20] Cohen P. Crawford T. N. Johnson J. G. Kasen S. (2005). The children in the community study of developmental course of personality disorder. *J. Pers. Disord.* 19 466–486. 10.1521/pedi.2005.19.5.466 16274277

[B21] Corbin W. R. Fromme K. (2002). Alcohol use and serial monogamy as risks for sexually transmitted diseases in young adults. *Health Psychol.* 21 229–236. 10.1037//0278-6133.21.3.229 12027028

[B22] Crandall A. Magnusson B. M. Novilla M. L. B. (2018). Growth in adolescent self-regulation and impact on sexual risk-taking: a curve-of-factors analysis. *J. Youth Adolesc.* 47 793–806. 10.1007/s10964-017-0706-4 28664311

[B23] Crick N. R. Murray–Close D. Woods K. (2005). Borderline personality features in childhood: a short-term longitudinal study. *Dev. Psychopathol.* 17 1051–1070. 10.1017/s0954579405050492 16613430

[B24] Dahl R. E. Allen N. B. Wilbrecht L. Suleiman A. B. (2018). Importance of investing in adolescence from a developmental science perspective. *Nature* 554 441–450. 10.1038/nature25770 29469094

[B25] Deardorff J. Gonzales N. A. Christopher F. S. Roosa M. W. Millsap R. E. (2005). Early Puberty and Adolescent Pregnancy: the Influence of Alcohol Use. *Pediatrics* 116 1451–1456. 10.1542/peds.2005-0542 16322170

[B26] Demos K. E. Healtherton T. F. Kelley W. M. (2012). Individual differences in nucleus accumbens activity to food and sexual images predict weight gain and sexual behavior. *J. Neurosci.* 32 5549–5552. 10.1523/JNEUROSCI.5958-11.2012 22514316 PMC3377379

[B27] Dir A. L. Coskunpinar A. Cyders M. A. (2014). A meta-analytic review of the relationship between adolescent risky sexual behavior and impulsivity across gender, age, and race. *Clin. Psychol. Rev.* 34 551–562. 10.1016/j.cpr.2014.08.004 25261740

[B28] Dunn S. Qi Xiong A. Nuernberger K. Norman W. V. (2019). Non-use of contraception by Canadian youth aged 15 to 24: findings from the 2009-2010 Canadian community health survey. *J. Obstetr. Gynaecol. Can.* 41 29–37. 10.1016/j.jogc.2018.05.021 30316712

[B29] Dziak J. J. Lanza S. T. Tan X. (2014). Effect size, statistical power and sample size requirements for the bootstrap likelihood ratio test in latent class analysis. *Struct. Equat. Model.* 21 534–552. 10.1080/10705511.2014.919819 25328371 PMC4196274

[B30] Edgardh K. (2000). Sexual behaviour and early coitarche in a national sample of 17 year old Swedish girls. *Sex. Trans. Infect.* 76 5–7. 10.1136/sti.76.2.98 PMC1758300 10858710

[B31] Edgardh K. (2007). Sexual behaviour and early coitarche in a national sample of 17-year-old Swedish boys. *Acta Psychiatri.* 91 985–991. 10.1111/j.1651-2227.2002.tb02889.x 12412877

[B32] Ensink K. Bégin M. Kotiuga J. Sharp C. Normandin L. (2019). Psychometric properties of the French version of the Borderline Personality Features Scale for Children and Adolescents. *Adolesc. Psychiatry* 9 1–11. 10.2174/2210676609666190820145256

[B33] Fetner T. Dion M. Heath M. Andrejek N. Newell S. L. Stick M. (2020). Condom use in penile-vaginal intercourse among Canadian adults: results from the sex in Canada survey. *PLoS One* 15:e0228981. 10.1371/journal.pone.0228981 32078662 PMC7032697

[B34] Finer L. B. (2010). Unintended pregnancy among U.S. adolescents: accounting for sexual activities. *J. Adolesc. Health* 47 312–314. 10.1016/j.jadohealth.2010.02.002 20708573

[B35] Fossati A. (2014). “Borderline personality disorder in adolescence: phenomenology and construct validy” in *Handbook of Borderline Personality Disorder in Children and Adolescents.* eds Sharp C. Tackett J. (New York: Springer). 19–34. 10.1007/978-1-4939-0591-1_3

[B36] Ge X. Conger R. D. Elder G. H. (2001). Pubertal transition, stressful life events, and the emergence of gender differences in adolescent depressive symptoms. *Dev. Psychol.* 37 404–417. 10.1037/0012-1649.37.3.404 11370915

[B37] Grant K. E. Stuhlmacher A. F. Thurm A. Mcmahon S. D. (2003). Stressors and child and adolescent psychopathology: moving from markers to mechanisms of risk. *Psychol. Bull.* 129 447–466. 10.1037/0033-2909.129.3.447 12784938

[B38] Harden K. P. (2014). A sex-positive framework for research on adolescent sexuality. *Perspect. Psychol. Sci.* 9 455–469. 10.1177/1745691614535934 26186753

[B39] Harned M. S. Pantalone D. W. Ward-Ciesielski E. F. Lynch T. R. Linehan M. M. (2011). The prevalence and correlates of sexual risk behaviors and sexually transmitted infections in outpatients with borderline personality disorder. *J. Nerv. Ment. Dis.* 199 832–8. 10.1097/NMD.0b013e318234c02c 22048134

[B40] Haslam N. Holland E. Kuppens P. (2012). Categories versus dimensions in personality and psychopathology: a quantitative review of taxometric research. *Psychol. Med.* 42 903–920. 10.1017/S0033291711001966 21939592

[B41] Hsu H. Wenzel S. Rice E. Gilreath T. D. Kurzban S. Unger J. (2015). Understanding consistent condom use among homeless men who have sex with women and engage in multiple sexual partnerships: a path analysis. *AIDS* 19 1676–1688. 10.1007/s10461-015-1051-9 25845531

[B42] Jardin C. Sharp C. Garey L. Vanwoerden S. Crist N. Elhai J. (2017). Compelled to risk: does sexual compulsivity explain the connection between borderline personality disorder features and number of sexual partners? *J. Pers. Disord.* 31 738–752. 10.1521/pedi_2017_31_277 28072043

[B43] Jenness S. M. Begier E. M. Neaigus A. Murrill C. S. Wendel T. Hagan H. (2011). Unprotected anal intercourse and sexually transmitted diseases in high-risk heterosexual women. *Am. J. Public Health* 101 745–750. 10.2105/AJPH.2009.181883 20558790 PMC3052332

[B44] Johnson J. G. Cohen P. Kasen S. Skodol A. E. Oldham J. M. (2008). Cumulative prevalence of personality disorders between adolescence and adulthood. *Acta Psychiatr. Scand.* 118 410–413. 10.1111/j.1600-0447.2008.01231.x 18644003

[B45] Kalichman S. C. Rompa D. (2001). Sexual Compulsivity Scale: further development and use with HIV-positive persons. *J. Pers. Assess.* 76 379–395. 10.1207/S15327752JPA7603_02 11499453

[B46] Kerpelman J. L. McElwain A. D. Pittman J. F. Adler-Baeder F. M. (2013). Engagement in risky sexual behavior: adolescents’ perceptions of self and the parent–child relationship matter. *Youth Soc.* 48 101–125. 10.1177/0044118X13479614

[B47] Kessler R. C. Berglund P. Demler M. A. Jin R. Merikangas K. R. Walters E. E. (2005). Lifetime prevalence and age-of-onset distributions of DSM-IV disorders in the national comorbidity survey replication. *Arch. Gener. Psychiatry* 62 593–602. 10.1001/archpsyc.62.6.593 15939837

[B48] Kim H. M. Miller L. C. (2020). Are insecure attachment styles related to risky sexual behavior? A meta-analysis. *Health Psychol.* 39 46–57. 10.1037/hea0000821 31724425

[B49] Laberge G. (2013). *Les Corrélats Intra et Interpersonnels de la Sexualité à Risque Chez les Adolescents et les Jeunes Adultes.* Trois-Rivières: Trois-Rivières Campus (Université du Québec à Trois-Rivières).

[B50] Lavan H. Johnson J. G. (2002). The association between axis I and II psychiatric symptoms and high-risk sexual behavior during adolescence. *J. Pers. Disord.* 16 73–94. 10.1521/pedi.16.1.73.22559 11881162

[B51] Leung S. W. Leung F. (2009). Construct validity and prevalence rate of borderline personality disorder among Chinese adolescents. *J. Pers. Disord.* 23 494–513. 10.1521/pedi.2009.23.5.494 19817630

[B52] Lewinsohn P. M. Rohde P. Seeley J. R. Klein D. N. (1997). Axis II psychopathology as a function of Axis I disorders in childhood and adolescence. *J. Am. Acad. Child Adolesc. Psychiatry* 36 1752–1759. 10.1097/00004583-199712000-00024 9401337

[B53] Lo Y. Mendell N. R. Rubin D. B. (2001). Testing the number of components in a normal mixture. *Biometrika* 88 767–778.

[B54] Macaluso M. Demand M. J. Artz L. M. Hook E. W. (2000). Partner type and condom use. *AIDS* 14 537–546. 10.1097/00002030-200003310-00009 10780716

[B55] Marcus D. K. Fulton J. J. Turchik J. A. (2011). Is risky sexual behavior continuous or categorical? A taxometric analysis of the Sexual Risk Survey. *Psychol. Assess.* 23 282–286. 10.1037/a0021842 21280955

[B56] Mendle J. Ferrero J. Moore S. R. Harden K. P. (2013). Depression and adolescent sexual activity in romantic and nonromantic relational contexts: a genetically-informative sibling comparison. *J. Abnorm. Psychol.* 122 51–63. 10.1037/a0029816 22985011

[B57] Miller A. L. Muehlenkamp J. J. Jacobson C. M. (2008). Fact or fiction: diagnosing borderline personality disorder in adolescents. *Clin. Psychol. Rev.* 28 969–981. 10.1016/j.cpr.2008.02.004 18358579

[B58] Moder K. (2007). How to keep the Type I error rate in ANOVA if variances are heteroscedastic. *Austr. J. Statist.* 36 179–188.

[B59] Moder K. (2010). Alternatives to F-test in one way ANOVA in case of heterogeneity of variances: a simulation study. *Psychol. Test Assess. Model.* 52 343–353.

[B60] Muthén L. K. Muthén B. O. (1998-2017). *Mplus User’s Guide Eighth Edition.* United States: National Institutes of Health.

[B61] Nylund K. L. Asparouhov T. Muthén B. O. (2007). Deciding on the number of classes in latent class analysis and growth mixture modeling: a Monte Carlo simulation study. *Struct. Equat. Model. Multidiscipl. J.* 14 535–569.

[B62] Parkes A. Wight D. Henderson M. Stephenson J. Strange V. (2009). Contraceptive method at first sexual intercourse and subsequent pregnancy risk: findings from a secondary analysis of 16-year-old girls from the RIPPLE and SHARE studies. *J. Adolesc. Health* 44 55–63. 10.1016/j.jadohealth.2008.06.006 19101459 PMC2606907

[B63] Partridge J. M. Koutsky L. A. (2006). Genital human papillomavirus infection in men. *Lancet Infect. Dis.* 6 21–31. 10.1016/S1473-3099(05)70323-6 16377531

[B64] Paus T. Keshavan M. Giedd J. N. (2008). Why do many psychiatric disorders emerge during adolescence? *Nat. Rev. Neurosci.* 9 947–957. 10.1038/nrn2513 19002191 PMC2762785

[B65] Penner F. Wall K. Jardin C. Brown J. L. Sales J. M. (2019). A Study of Risky Sexual Behavior, Beliefs About Sexual Behavior, and Sexual Self-Efficacy in Adolescent Inpatients With and Without Borderline Personality Disorder. *Pers. Disord. Theor. Res. Treat.* 10 524–535. 10.1037/per0000348 31259605

[B66] Ramaswamy V. Desarbo W. S. Reibstein D. J. Robinson W. T. (1993). An empirical pooling approach for estimating marketing mix elasticities with PIMS date. *Market. Sci.* 12 103–124.

[B67] Reid R. C. Garos S. Carpenter B. N. (2011). Reliability, validity, and psychometric development of the Hypersexual Behavior Inventory in an outpatient sample of men. *Sex. Addict. Compuls.* 18 30–51.

[B68] Rickards S. Laaser M. (1999). Sexual actingout in borderline women: impulsive self-destructiveness or sexual addiction/compulsivity? *Sex. Addict. Compuls.* 6 31–45. 10.1080/10720169908400177

[B69] Roterman M. (2012). Sexual behaviour and condom use of 15-to 24-year-olds in 2003 and 2009/2010. *Health Rep.* 3 41–5. 22590804

[B70] Rothschild L. Cleland C. Haslam N. Zimmerman M. (2013). A taxometric study of borderline personality disorder. *J. Abnorm. Psychol.* 112 657–666. 10.1037/0021-843X.112.4.657 14674877

[B71] Sansone R. A. Chu J. W. Wiederman M. W. (2011a). Sexual behaviour and borderline personality disorder among female psychiatric inpatients. *Int. J. Psychiatry Clin. Pract.* 15 69–73. 10.3109/13651501.2010.507871 22122692

[B72] Sansone R. A. Lam C. Wiederman M. W. (2011b). The relationship between borderline personality disorder and number of sexual partners. *J. Pers. Disord.* 25 782–788. 10.1521/pedi.2011.25.6.782 22217224

[B73] Sansone R. A. Sansone L. A. (2011). Sexual behavior in borderline personality: a review. *Innovat. Clin. Neurosci.* 8 14–18. PMC3071095 21468292

[B74] Sansone R. A. Wiederman M. W. (2009). Borderline personality symptomatology, casual sexual relationships, and promiscuity. *Psychiatry* 6 36–40. PMC2719454 19724753

[B75] Schwarz G. (1978). Estimating the dimension of a model. *Ann. Statist.* 6 461–464.

[B76] Shannon C. L. Klausner J. (2018). The growing epidemic of sexually transmitted infections in adolescents: a neglected population. *Curr. Opin. Pediatrics* 30 137–143. 10.1097/MOP.0000000000000578 29315111 PMC5856484

[B77] Sharp C. (2016a). Bridging the gap: the assessment and treatment of adolescent personality disorder in routine clinical care. *Arch. Dis. Childh.* 102 103–108. 27507846 10.1136/archdischild-2015-310072

[B78] Sharp C. (2016b). Current trends in BPD research as indicative of a broader sea-change in psychiatric nosology. *Pers. Disord.* 7 334–343. 10.1037/per0000199 27709990

[B79] Sharp C. Fonagy P. (2015). Practionner review: borderline personality disorder in adolescence-Recent conceptualization, intervention and implications for clinical practice. *J. Child Psychol. Psychiatry* 56 1266–1288. 26251037 10.1111/jcpp.12449

[B80] Sharp C. Mosko O. Chang B. Ha C. (2011). The cross-informant concordance and concurrent validity of the Borderline Personality Features Scale for Children in a community sample of boys. *Clin. Child Psychol. Psychiatry* 16 335–349. 10.1177/1359104510366279 20921039

[B81] Sisk C. L. Zehr J. L. (2005). Pubertal hormones organize the adolescent brain and behavior. *Front. Neuroendocrinol.* 26 163–174. 10.1016/j.yfrne.2005.10.003 16309736

[B82] Stepp S. D. (2012). Development of borderline personality disorder in adolescence and young adulthood: introduction to the special section. *J. Abnorm. Child Psychol.* 40 1–5. 10.1007/s10802-011-9594-3 22116635 PMC3865353

[B83] Stepp S. D. Keenan K. Hipwell A. E. Krueger R. F. (2014). The impact of childhood temperament on the development of borderline personality disorder symptoms over the course of adolescence. *Borderline Pers. Disord. Emot. Dysregul.* 1 18–18. 10.1186/2051-6673-1-18 26064524 PMC4459747

[B84] Stöckl H. March L. Pallitto C. Garcia-Moreno C. (2014). Intimate partner violence among adolescents and young women: prevalence and associated factors in nine countries: a cross-sectional study. *BMC Public Health* 14:751. 10.1186/1471-2458-14-751 25059423 PMC4133076

[B85] Suleiman A. B. Galvàn A. Harden K. P. Dahl R. E. (2017). Becoming a sexual being: the ‘elephant in the room’ of adolescent brain development. *Dev. Cogn. Neurosci.* 25 209–220. 10.1016/j.dcn.2016.09.004 27720399 PMC6987766

[B86] Thompson K. N. Betts J. Jovev M. Nyathi Y. McDougall E. Chanen A. M. (2017). Sexuality and sexual health among female youth with borderline personality disorder pathology. *Early Intervent. Psychiatry* 13 502–508. 10.1111/eip.12510 29076247

[B87] Turchik J. A. Garske J. P. (2009). Measurement of Sexual Risk Taking Among College Students. *Arch. Sex. Behav.* 38 936–948. 10.1007/s10508-008-9388-z 18563548

[B88] Turner R. J. Lloyd D. A. (2004). Stress burden and the lifetime incidence of psychiatric disorder in young adults: racial and ethnic contrasts. *Arch. Gener. Psychiatry* 61 481–488. 10.1001/archpsyc.61.5.481 15123493

[B89] Victor E. C. Hariri A. R. (2016). A neuroscience perspective on sexual risk behavior in adolescence and emerging adulthood. *Dev. Psychopathol.* 28 471–487. 10.1017/S0954579415001042 26611719 PMC4828296

[B90] Weinstock H. Berman S. Cates W. (2004). Sexually transmitted diseases among American Youth: incidence and prevalence estimates, 2000. *Perspect. Sex. Reprod. Health* 36 6–10. 10.1363/3600604 14982671

[B91] Winsper C. Lereya S. T. Marwaha S. Thompson A. Eyden J. Singh S. P. (2016). The aetiological and psychopathological validity of borderline personality disorder in youth: a systematic review and meta-analysis. *Clin. Psychol. Rev.* 44 13–24. 10.1016/j.cpr.2015.12.001 26709502

[B92] Yoon S. Voith L. A. Kobulsky J. M. (2018). Gender difference in pathways from child physical and sexual abuse to adolescent risky sexual behavior among high-risk youth. *J. Adolesc.* 64 89–97. 10.1016/j.adolescence.2018.02.006 29438874

[B93] Zanarini M. C. Horwood L. J. Wolke D. Waylen A. Fitzmaurice G. Grant B. F. (2011). Prevalence of DSM-IV borderline personality disorder in two community samples: 6,330 English 11-year-olds and 34,653 American adults. *J. Pers. Disord.* 25 607–619. 10.1521/pedi.2011.25.5.607 22023298 PMC4678770

